# LGR5 promotes epithelial ovarian cancer proliferation, metastasis, and epithelial–mesenchymal transition through the Notch1 signaling pathway

**DOI:** 10.1002/cam4.1485

**Published:** 2018-05-18

**Authors:** Wenxue Liu, Jing Zhang, Xupei Gan, Fangqian Shen, Xiaoming Yang, Na Du, Dandan Xia, Lei Liu, Lianqiao Qiao, Jufang Pan, Yunyan Sun, Xiaowei Xi

**Affiliations:** ^1^ Department of Obstetrics and Gynecology Shanghai General Hospital School of Medicine Shanghai Jiao Tong University Shanghai 200080 China; ^2^ Department of Obstetrics and Gynecology The International Peace Maternity & Child Health Hospital School of Medicine Shanghai Jiao Tong University Shanghai 200080 China; ^3^ Department of Obstetrics and Gynecology Ren Ji Hospital School of Medicine Shanghai Jiao Tong University Shanghai 200127 China

**Keywords:** Epithelial–mesenchymal transition, epithelial ovarian cancer, LGR5, metastasis, Notch1 signaling pathway

## Abstract

Leucine‐rich repeat‐containing G protein‐coupled receptor 5 (LGR5) plays a vital role in the development of malignant tumors; however, its biological role and underlying mechanism in epithelial ovarian cancer (EOC) remain unclear. In this study, we aimed to investigate the biological function and clinical significance of LGR5 in human EOC. We evaluated LGR5 expression in EOC cell lines and tissues from ovarian cancer patients by qPCR, Western blotting, and immunohistochemical analysis. Cell proliferation, colony formation, transwell invasion assay, and scratch‐wound assays were conducted to evaluate the expansion and invasion abilities of EOC cells. Tumor xenograft experiments were performed in female BALB/c athymic nude mice to test cell proliferation in vivo. Western blot analysis was performed to confirm the expression of epithelial‐to‐mesenchymal transition (EMT) signature proteins and their association with Notch1 signaling. The results demonstrated that LGR5 was overexpressed in EOC tissues and cell lines. Aberrant expression of LGR5 was significantly associated with patient age (*P* = 0.006), tumor histologic type (*P* < 0.001), and distant metastasis (*P* = 0.025). Consistent with these findings, suppression of LGR5 expression led to decreased proliferation and metastasis of EOC cell lines. Furthermore, LGR5 could induce EMT and regulate the Notch1 signaling pathway. Taken together,LGR5 may have an important role in the promotion of tumorigenesis and metastasis of EOC and is a potential therapeutic target for EOC management.

## Introduction

Epithelial ovarian cancer (EOC) is the most common histological type of ovarian cancer, accounting for 80% to 90% of malignancies. EOC is also the most lethal of malignant tumors of the female reproductive system 1. The prognosis for patients suffering from ovarian cancer remains poor because of limited therapeutic strategies and late diagnosis. It is estimated that over 70% of patients are diagnosed at an advanced stage of disease, and only approximately 30% of patients survive for 5 years or more 2. The detailed pathogenesis of EOC remains elusive. Thus, study of the molecular mechanisms underlying EOC and the search for promising novel therapeutic strategies will be indispensable for improvement of the prognosis of patients with EOC.

LGR5 (also known as GPR49, HG38, or FEX) belongs to the glycoprotein hormone receptor subfamily comprising proteins with seven transmembrane domains. Typically, these proteins are structurally similar to glycoprotein hormone receptors, including thyroid‐stimulating hormone, follicle‐stimulating hormone, and luteinizing hormone receptors 3. LGR5 has a large N‐terminal extracellular domain‐containing 17 leucine‐rich repeats that are important for interaction with its glycoprotein ligands 4, 5. LGR5 has been identified as a novel marker of adult stem cells in the small intestine, hair follicles, and gastric corpus epithelium 6, 7, 8 and is widely expressed in spinal cord, breast, hair follicle, and brain tissues 4. LGR5 also plays an important role during embryogenesis. It has been reported that LGR5 is overexpressed in ovarian cancer 9; however, its role in cell proliferation, migration, and invasion, and the underlying molecular mechanisms remain unclear.

The Notch signaling pathway is highly conserved through evolution; comprises receptors, ligands, and intracellular proteins; and plays a critical role in cell proliferation, differentiation, development, and homeostasis 10. There are four members of the mammalian Notch gene family, Notch1–4 11. Previous studies have implied a role for aberrant Notch1 signaling pathway in tumorigenesis of various malignancies, including pancreatic cancer 12, prostate cancer 13, and melanoma 14. Epithelial–mesenchymal transition (EMT) is defined as a reversible process in which epithelial cells acquire the mesenchymal phenotype 15. EMT plays a key role in embryonic development, tissue regeneration, and cancer metastasis 16. Various signaling pathways induce EMT, including the HGF, EGF, TGF‐*β*, Wnt/*β*‐catenin, and Notch signaling pathways 17. As an important regulator of EMT, activation of the Notch1 pathway is common in numerous malignant tumors including EOC 18. Furthermore, it is proposed that Notch signaling is activated in LGR5‐positive cells from intestinal tumors of APC‐deficient mice 19. Therefore, we speculated that LGR5 may promote the development of EOC through regulation of the Notch1 signaling pathway.

In this study, we investigated the expression of LGR5 at the mRNA and protein levels in human ovarian cancer and verified correlations between LGR5 expression and clinicopathological parameters. We demonstrated that LGR5 can promote cell proliferation and migration in vitro. Moreover, we also determined that LGR5 could induce EMT via the Notch1 pathway in EOC. Our findings indicate that LGR5 functions as a novel tumor oncogene in EOC, and may be a potential therapeutic target.

## Materials and Methods

### Immunohistochemistry

For immunohistochemistry, a tissue array comprising sections from 93 ovarian cancer cases and five normal ovarian samples (Zhuo Li Biotech, Shanghai, China) was deparaffinized in xylene and rehydrated through descending concentrations of ethanol. We also collected 25 normal samples from Shanghai General Hospital, and our experiment was approved by the Institutional Research Ethics Committee of Shanghai General Hospital. Then, an antigen retrieval process was performed using 0.01 mol/L sodium citrate buffer (pH 6.0). After the sections were blocked with 3% hydrogen peroxide, the expression of LGR5 was assessed using standard immunohistochemical methods 20 with primary antibody against LGR5 (Abcam, Cambridge, UK; 1:100 dilution). The intensity and extent of staining were independently evaluated by two pathologists who were blinded to patient information. Staining intensity was graded as follows: 0, no staining; 1, mild staining; 2, moderate staining; and 3, intense staining. The staining area was scored as follows: 0, no staining of cells; and 1, 1–25%; 2, 26–50%; 3, 51–75%; and 4, 76–100% of cells stained. A sum of staining scores (intensity and extension) index was used as the final staining score, graded as follows: 0–1, negative; 2–4, weakly positive; and 5–7, strongly positive expression, where both weakly positive and strongly positive samples were considered as positive for LGR5 expression.

### Western blotting

Treated cells were lysed on ice in RIPA buffer containing 1 mmol/L phenylmethylsulfonyl fluoride. Cell lysate samples containing approximately 30 *μ*g of protein were loaded into the wells of 7.5–12% sodium dodecyl sulfate–polyacrylamide gels electrophoresis carried out for 1–2 h at 80 V, and proteins were transferred to PVDF membranes (Millipore, Bedford, MA). Membranes were then blocked in 5% skim milk for 1 h at room temperature, followed by incubation overnight at 4°C with specific primary antibodies (1:1000 dilution). After washing with TBST buffer three times (10 min each), membranes were incubated with appropriate secondary antibodies conjugated to horseradish peroxidase (1:5000 dilution; Proteintech, Chicago, IL) at room temperature for 1 h. Proteins were then detected using enhanced chemiluminescence reagents (Pierce, Rockford, IL). The primary antibodies used in our study included LGR5 (Abcam, Cambridge, UK), cyclin‐D1, c‐Myc (Proteintech), E‐cadherin, N‐cadherin, Snail, Notch1 (Cell Signaling Technology, Danvers, MA), and HES1 (Cell Signaling Technology). GAPDH (Proteintech) was used as a loading control. Each experiment was performed in triplicate.

### Transient transfection

Cells were transiently transfected using Lipofectamine 3000 (Invitrogen, Grand Island, NY) in accordance with the manufacturer's advised procedure. Briefly, cells were seeded into 6‐well plates at a density of 2 × 10^4 ^cells/well. After culture to 50%–60% confluency, cells were serum starved for 24 h to minimize the influence of FBS. Then, cells were transfected with siRNA or plasmids using Lipofectamine 3000. After 6–8 h of incubation, treated cells were cultured in DMEM/F‐12 with 10% FBS. LGR5 and negative control siRNAs were constructed by GeneChen (Shanghai, China). The plasmids pcDNA3.1‐LGR5 and pcDNA3.1 were designed and purchased from Genera Biotechnology (Shanghai, China). The sequences of the LGR5 siRNAs were as follows:

sense, 5′‐CCUAGAGACUUUAGAUUUAdTdT‐3′;

antisense, 5′‐UAAAUCUAAAGUCUCUAGGdTdT‐3′.

### RNA extraction and quantitative real‐time PCR (qPCR)

Samples from four cell lines were analyzed by qPCR. Total RNA was extracted using Trizol reagent (TaKaRa, Japan), according to the manufacturer's instructions. LGR5 expression levels were assessed using 2 *μ*L of cDNA and the following primer sequences: forward, 5′‐GATGACCATTGCCTACACC‐3′ and the antisense primer sequence was 5′‐ACCATAGAGCAGTCCCAAAT‐3′. GAPDH served as an internal control and the following primer sequences: forward, 5′‐GGG AAG GTG AAG GTC GGA GT‐3′ and the antisense primer sequences was 5′‐GGG GTC ATT GAT GGC AAC A‐3′. All experiments were performed in triplicate.

### Cell proliferation and plate colony formation assays

Cells were seeded into 96‐well plates (2000 cells per well) and cultured for 24 h. Subsequently, 10 *μ*L Cell Counting Kit‐8 reagent (CCK‐8; Dojindo Laboratories, Japan) in 100 *μ*L medium was added per well for 1 h at 37°C, and then, absorbance was measured at 450 nm to evaluate cell growth. For in vitro colony formation assays, 500 transfected cells were seeded in 6‐well plates and cultured in conditioned medium at 37°C for 2 weeks. After fixing with methanol and staining with crystal violet (Beyotime, Shanghai, China), colonies were counted and photographed. All experiments were performed in triplicate.

### Cell invasion assays

Cell migration and invasion assays were performed using transwell plates with 8‐*μ*m‐pore filters (Corning, NY), following the manufacturer's protocol. Briefly, for the cell migration assay, transfected Hey, SKOV3, and HO8910 cells (2 × 10^5^) were suspended in 200 *μ*L of serum‐free medium and seeded into the upper chambers of the transwell plates, and medium supplemented with 20% fetal bovine serum was applied to the lower chamber as a chemoattractant to induce migration. After incubation for 12–24 h at 37°C, tumor cells were fixed with 4% cold paraformaldehyde and stained with crystal violet (Beyotime, Shanghai, China). For the invasion assay, the procedures were conducted as described above, except that filter inserts were coated with BD Matrigel and the plates were cultured for 16–24 h at 37°C. Migrated cells were counted at 100× magnification under an inverted microscope. Independent experiments were performed in triplicate.

### Cell scratch‐wound assay

Treated cells were seeded into 6‐well plates and cultured until confluence. A wound was generated by scraping with a 10‐*μ*L pipette tip. After 24 h, the cells in the wounded monolayer were photographed and cell migration was assessed by measuring gap sizes in multiple fields.

### Animal experiments

Female BALB/c athymic nude mice (4–6 weeks old) were purchased from the Institute of Zoology, Chinese Academy of Sciences of Shanghai. Three mice were the experimental group, and three mice were used for control. All mice were injected subcutaneously with 2 × 10^6^ SKOV3 cells stably transfected with an LGR5 expression vector or a control vector. The total weights of their tumor nodules were measured and recorded. The tumor volume was calculated using the formula: length × width^2 ^× 0.5. All mice were euthanized 4 weeks after tumor cell injection and subjected to autopsy. All of the animal procedures were conducted in accordance with the Shanghai Jiaotong University Affiliated Shanghai First People's Hospital Animal Care guidelines. All efforts were made to minimize animal suffering.

### Statistical analyses

Statistical analyses were performed using SPSS ver. 20.0 (IBM, Armonk, NY). Data are presented as means ± standard deviation (SD). Differences between test and control groups were evaluated using Student's *t*‐tests. *P* values <0.05 were considered statistically significant.

## Results

### LGR5 is overexpressed in EOC tumor tissues and some cell lines

**Figure 1:**
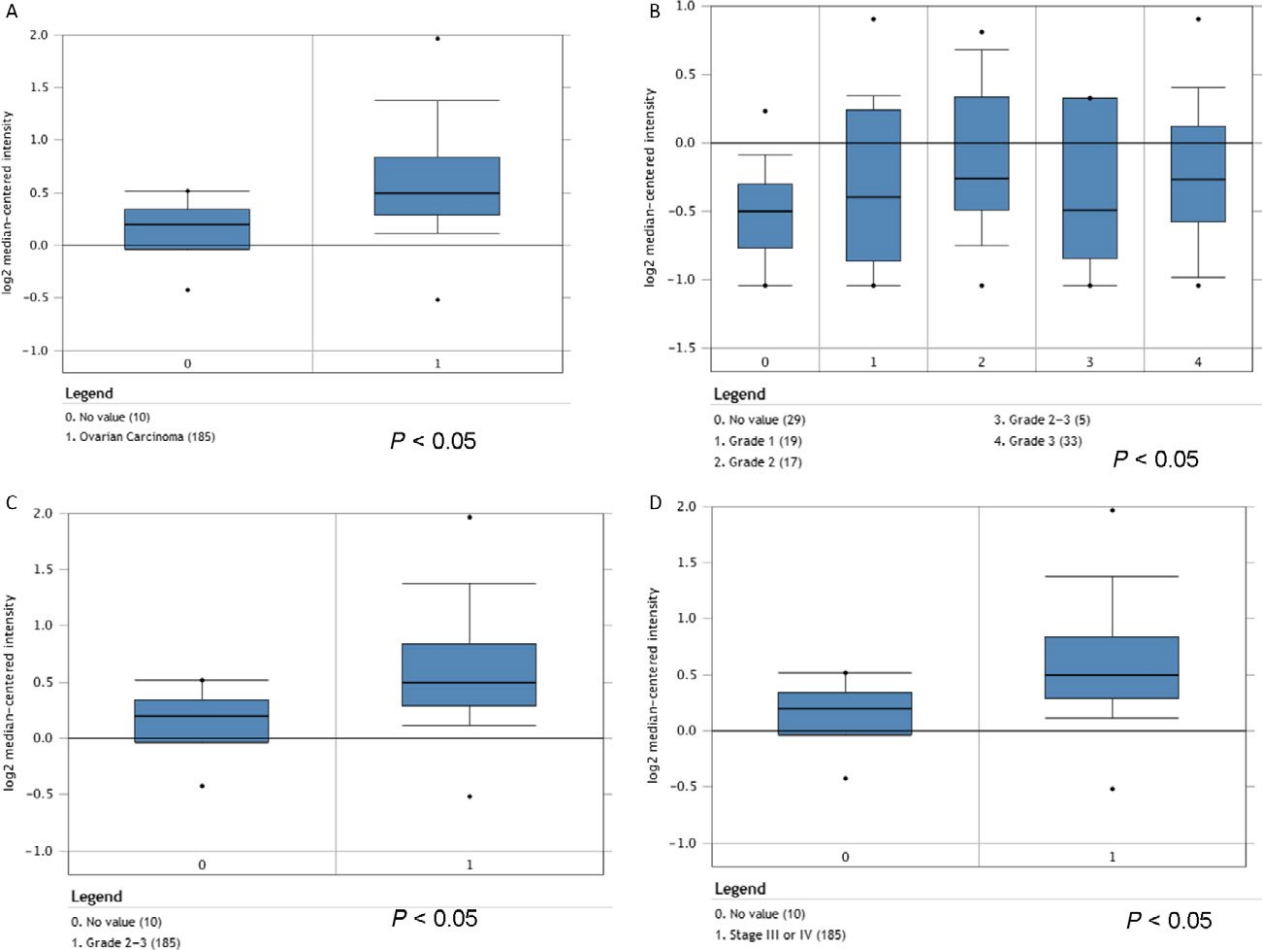
Analysis of LGR5 RNA expression based on Oncomine data. (A) LGR5 expression of normal specimens and ovarian carcinoma demonstrating that LGR5 expression levels were increased in ovarian cancer relative to control samples (Cancer Res 2008/07/01). *P* = 0.0001. (B–C) LGR5 expression levels were increased in grades 1, 2, 3 (Cancer Res 2006/02/01), and 2–3 (Cancer Res 2008/07/01) tumors. *P* = 0.0001. (D) LGR5 expression levels were increased in stage III or IV tumors compared with normal tissue (Cancer Res 2008/07/01). *P* = 0.0001.

**Figure 2:**
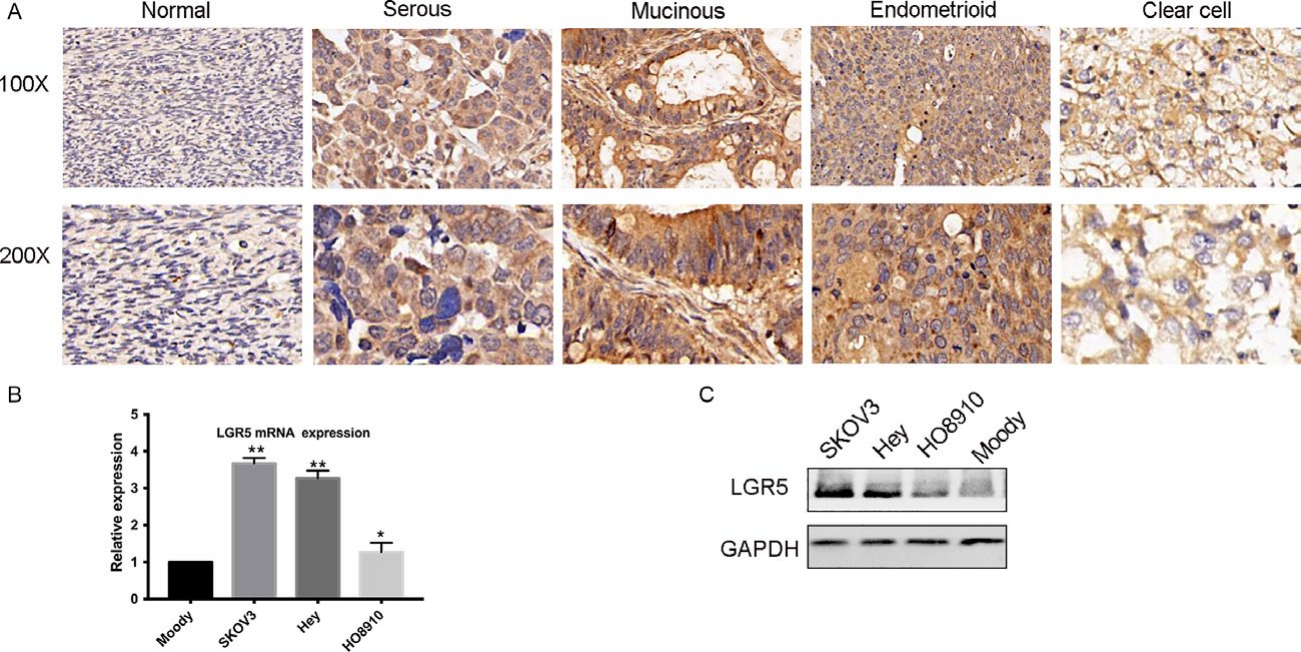
LGR5 expression and pathologic features in EOC specimens. (A) Immunohistochemical staining of LGR5. LGR5 was overexpressed and localized within the cytoplasm of tumor cells in EOC tissue specimens (scale bar = 20 *μ*m). (B–C) qPCR and Western blot analysis were performed in three EOC cell lines to assess LGR5 expression levels. The human ovarian epithelial cell line, Moody, was used as control.

To identify the role of LGR5 in ovarian cancer, we first browsed the Oncomine (http://www.oncomine.org) database of cancer transcriptome profiles and found that the gene expression levels of LGR5 were significantly higher in tumor compared with related normal tissues (Fig. 1A) and closely correlated with tumor grade (Fig. 1B and C). Moreover, Oncomine LGR5 gene expression data from patients obtained in RNA‐Seq experiments showed that the expression levels of LGR5 in ovarian cancer patients were augmented in stages III and IV, relative to that in normal tissues (Fig. 1D). To further confirm that LGR5 overexpression is associated with ovarian carcinogenesis, immunohistochemistry was used to analyze 93 samples of randomly selected cancer tissues (representative images, Fig. 2A). Immunohistochemical analysis showed that 84.9% (79/93) of EOC tissues showed intense staining for LGR5 (Table 1). Upregulated expression of LGR5 was significantly correlated with patient age (≥60 years; *P* = 0.026), histologic type (*P* < 0.001), and metastasis (*P* = 0.025) in EOC (Table 2). Next, we assessed LGR5 expression levels by qPCR and Western blotting in three ovarian cancer cell lines (SKOV3, Hey, HO8910). LGR5 overexpression was detected in three epithelial ovarian cancer lines (SKOV3, Hey, and HO8910) examined compared with the human ovarian epithelial cell line, Moody (Fig. 2B andC).

**Table 1:** Expression of LGR5 in epithelial ovarian cancer (EOC) compared to normal ovarian epithelial tissue (SD)

Protein expression (*n*)				
Tissue	Total	High	Low	*P* value
Normal	30	2	28	<0.01
EOC	93	14	79	

**Table 2:** The association between LGR5 expression and the clinicopathologic characteristics of patients with epithelial ovarian cancer (SD)

LGR5 staining				
Characteristics	No. of patients	Low	High	*P* value
Age (years)				
≥60	77	8	69	0.026*
<60	16	6	10	
Histologic type				
Serous	48	3	45	
Mucinous	15	3	12	<0.001**
Endometriosis	29	7	22	
Clear cell	1	0	1	
Normal	30		2	
FIGO stage				
I–II	72	9	63	0.918
III–IV	21	5	16	
N stage				
N0	87	12	75	0.084
N1	6	1	5	
M stage				
M0	74	8	66	0.025*
M1	19	6	13	

*P < 0.05, **P < 0.001

### LGR5 promotes the proliferation of EOC cells

**Figure 3:**
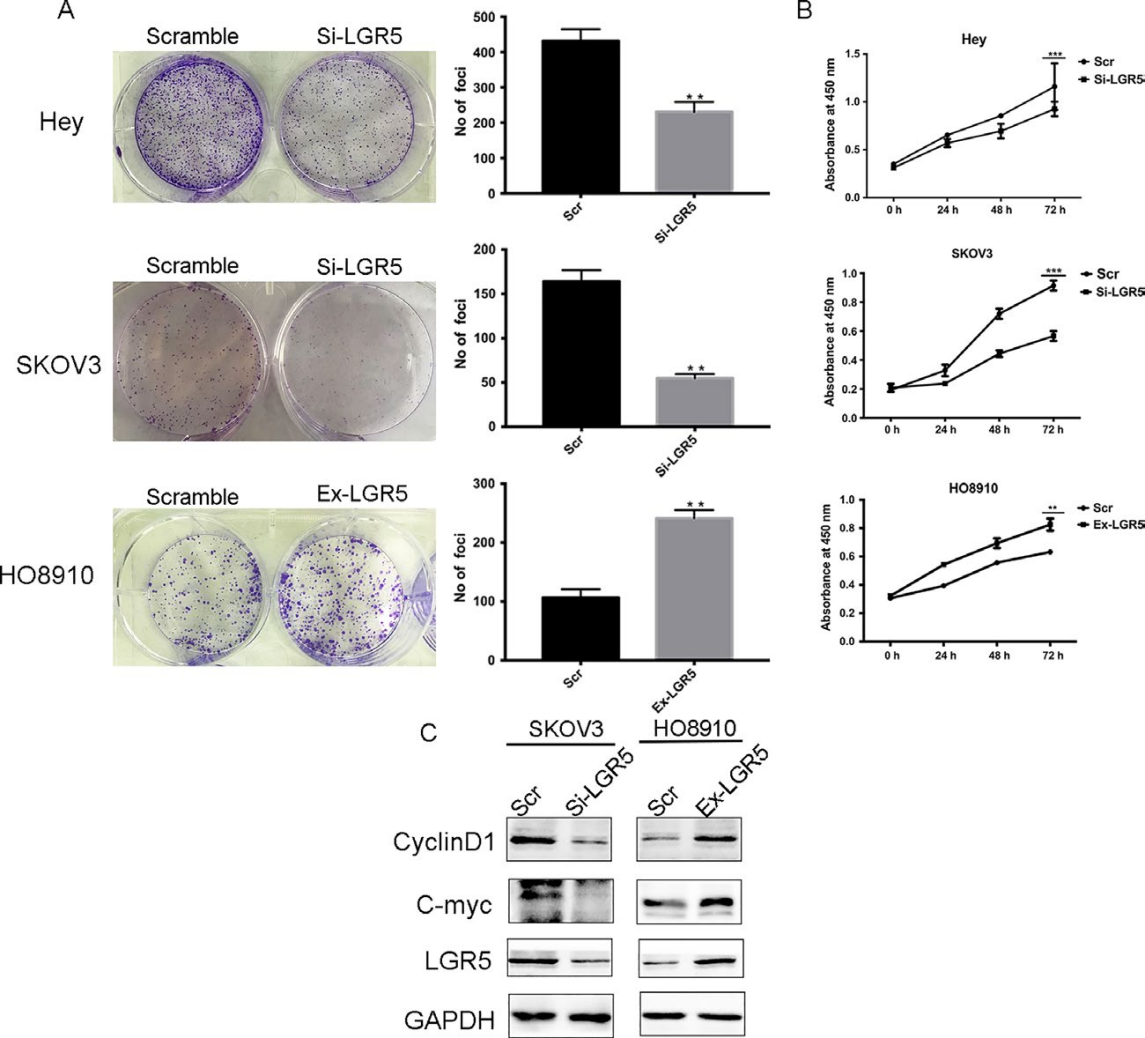
Elevated expression of LGR5 promotes the proliferation of EOC cells in vitro. (A) LGR5 siRNA (Si‐LGR5) inhibited EOC (Hey and SKOV3 cells) colony formation in vitro, while overexpression of LGR5 in HO8910 cells (Ex‐LGR5) enhanced EOC cell colony formation in contrast with the control (Scramble). (B) Proliferation of Hey, SKOV3, and HO8910 cells treated with LGR5 siRNA (Si‐LGR5) or with LGR5 overexpression (Ex‐LGR5) and their respective controls were evaluated using CCK‐8 assays. (C) Compared to the control group, knockdown of LGR5 (Si‐LGR5) in SKOV3 cells inhibited the expression of cyclin D1 and C‐myc, in contrast to the effects of LGR5 overexpression in HO8910 cells.

To uncover the potential functions of LGR5 in EOC tumorigenesis, small interfering RNA (siRNA) was transfected into SKOV3 or Hey cells to silence LGR5 expression. A colony formation assay demonstrated that LGR5 could increase the number of foci formed by ovarian cancer cells and promote tumor growth (Fig. 3A). Then, cell growth assays were performed using a CCK8 kit (Fig. 3B). The resulting growth curves demonstrated that knockdown of LGR5 in both Hey and SKOV3 cells significantly inhibited cell proliferation, compared with their negative controls (Fig. 3B); however, LGR5 overexpression markedly promoted growth of HO8910 cells (Fig. 3A; *P* < 0.01). These results demonstrate that LGR5 can promote the proliferation of ovarian cancer cells. Moreover, the expression of proliferation‐related proteins (cyclin D1 and C‐myc) were detected by Western blot analysis. As shown in Figure 3C, compared with the control group, knockdown of LGR5 in SKOV3 cells led to inhibition of the expression of cyclin D1 and C‐myc, in contrast to LGR5 overexpression in HO8910 cells.

### LGR5 facilitates invasion and metastasis of EOC cells in vitro

**Figure 4:**
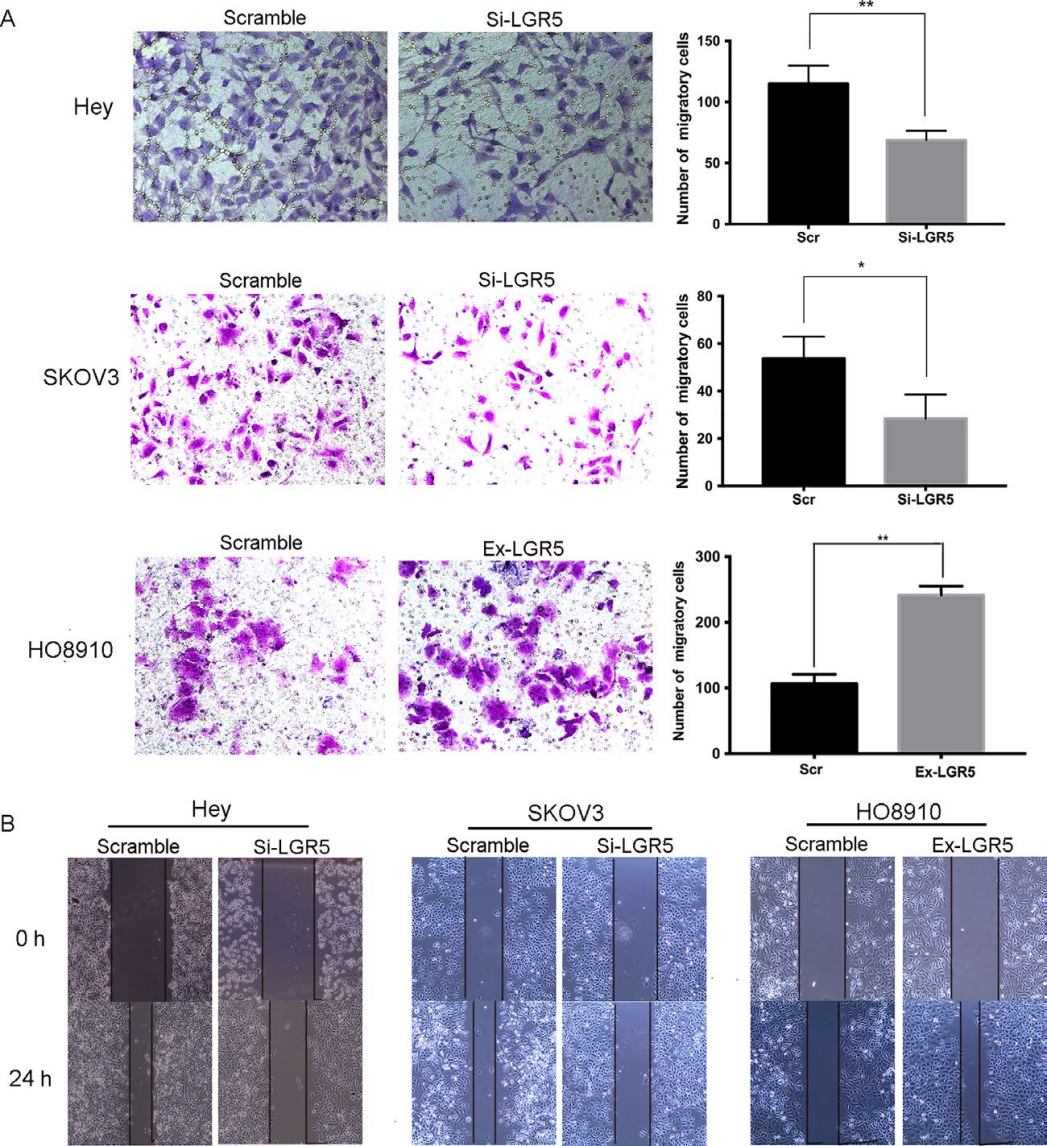
LGR5 promotes the migration and invasion capacity of ovarian cancer cells through EMT. (A) Transwell migration assays of Hey and SKOV3 cells treated with LGR5 siRNA (Si‐LGR5), HO8910 cells overexpressing LGR5 (Ex‐LGR5), and their respective controls. Quantification of cells that migrated through the membrane (right) was performed using data from three randomly selected fields of view. Original magnification 200×. Data are presented as means ± SD. *P < 0.05, **P < 0.01, compared with the control group. (B) Wound‐healing migration assays of Hey and SKOV3 cells treated with LGR5 siRNA (Si‐LGR5), HO8910 cells overexpressing LGR5 (Ex‐LGR5), and their respective controls (Scramble). Knockdown of LGR5 in SKOV3 and Hey cells resulted in reduced wound‐healing ability, compared with control cells, which was restored by increase in LGR5 expression in HO8910 cells. Original magnification 200×, scale bar = 20 *μ*m.

To further evaluate the potential mechanism of action of LGR5 in the tumorigenesis of EOC, we next studied the impact of LGR5 on cell migration and invasion in vitro. The results of transwell invasion assays revealed that silencing LGR5 remarkably reduced the number of cells on membrane filters compared with controls, while overexpression of LGR5 increased the number of cells present (*P* < 0.05, Fig. 4A). Moreover, scratch‐wound‐healing assays also demonstrated that knockdown of LGR5 in SKOV3 and Hey cells resulted in reduced wound‐healing ability, compared with control cells, which was restored by increase in LGR5 expression in HO8910 cells (Fig. 4B). Collectively, these results suggest that LGR5 contributes to the migration and invasion capacity of EOC cells.

### LGR5 induces EMT in epithelial ovarian cancer

**Figure 5:**
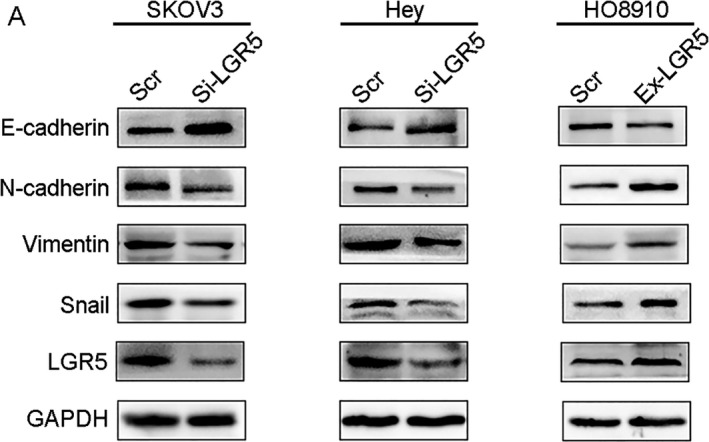
Effect of LRG5 on expression of epithelial‐to‐mesenchymal transition markers. (A) Western blots confirmed that E‐cadherin was upregulated while N‐cadherin, Vimentin, and Snail were downregulated in Hey and SKOV3 cells treated with LGR5 siRNA (Si‐LGR5), in contrast with HO8910 cells overexpressing LGR5 (Ex‐LGR5).

For the majority of carcinomas, progression toward malignancy is accompanied by loss of epithelial differentiation and a shift toward a mesenchymal phenotype. This process is referred to as EMT 21. A previous study showed that LGR5 is involved in breast cancer progression 22. Hence, to elucidate whether LGR5 has a role in inducing EMT in EOC, we examined the expression of EMT‐related proteins by Western blot analysis. As shown in Figure 5A, the expression of the mesenchymal markers, N‐cadherin and Vimentin, was inhibited in Hey and SKOV3 cells transfected with siRNA silencing LGR5 compared with controls, as was that of the EMT‐related transcription factor, Snail. The expression of the epithelial marker, E‐cadherin, was stimulated in LGR5 siRNA‐treated cells, with the opposite effect in HO8910 cells overexpressing LGR5. These results indicate that LGR5 promotes migration and invasion through promotion of EMT in vitro.

### LGR5 could activate the Notch1 signaling pathway in EOC

**Figure 6:**
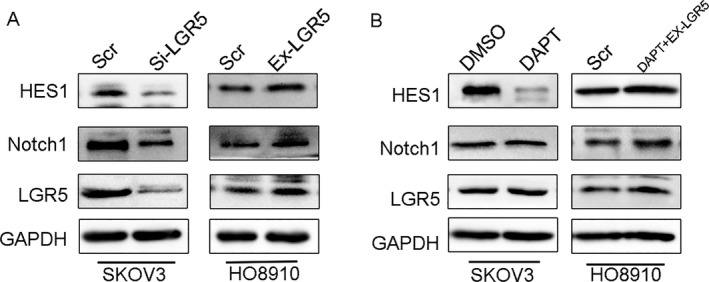
LGR5 can activate the Notch1 signaling pathway. (A) Western blot analysis showed that silencing of LGR5 (Si‐LGR5) expression in SKOV3 cells decreased Notch1 expression levels, while increased LGR5 expression (Ex‐LGR5) in HO8910 cells upregulated the expression of Notch1. (B) Western blotting analysis in HO8910 cells with LGR5 overexpression and treatment with 10 *μ*mol/L DAPT.

The Notch pathway has a powerful influence on stem cell maintenance, development, and cell fate and is increasingly recognized for the key roles it plays in cancer. Notch promotes cell survival, angiogenesis, and treatment resistance in numerous cancers, making it a promising target for cancer therapy 22, 23. Owing to the fundamental role of Notch1 signaling in tumorigenesis and metastasis of tumors, we wondered whether LGR5 participated in EOC progression via the Notch1 signaling pathway. As shown in Figure 6A, transfection with LGR5‐siRNA significantly reduced the expression of Notch1 and its target gene HES1. In contrast, overexpression of LGR5 led to increased expression of Notch1 and HES1. To further investigate the effect of Notch activation in EOC cells, we pretreated HO8910 cells with 10 *μ*mol/L DAPT (a *γ*‐secretase inhibitor to inactivate Notch1 pathway) or an equal volume of DMSO (the vehicle control) for 24 h. Western blotting analysis showed that inhibition of Notch by DAPT did not affect the expression of LGR5. We also found that simultaneous DAPT‐mediated downregulation of HES1 could be restored by overexpressing LGR5. Taken together, LGR5 may regulate the Notch1 signaling pathway in EOC.

### LGR5 promotes tumorigenesis in vivo

**Figure 7:**
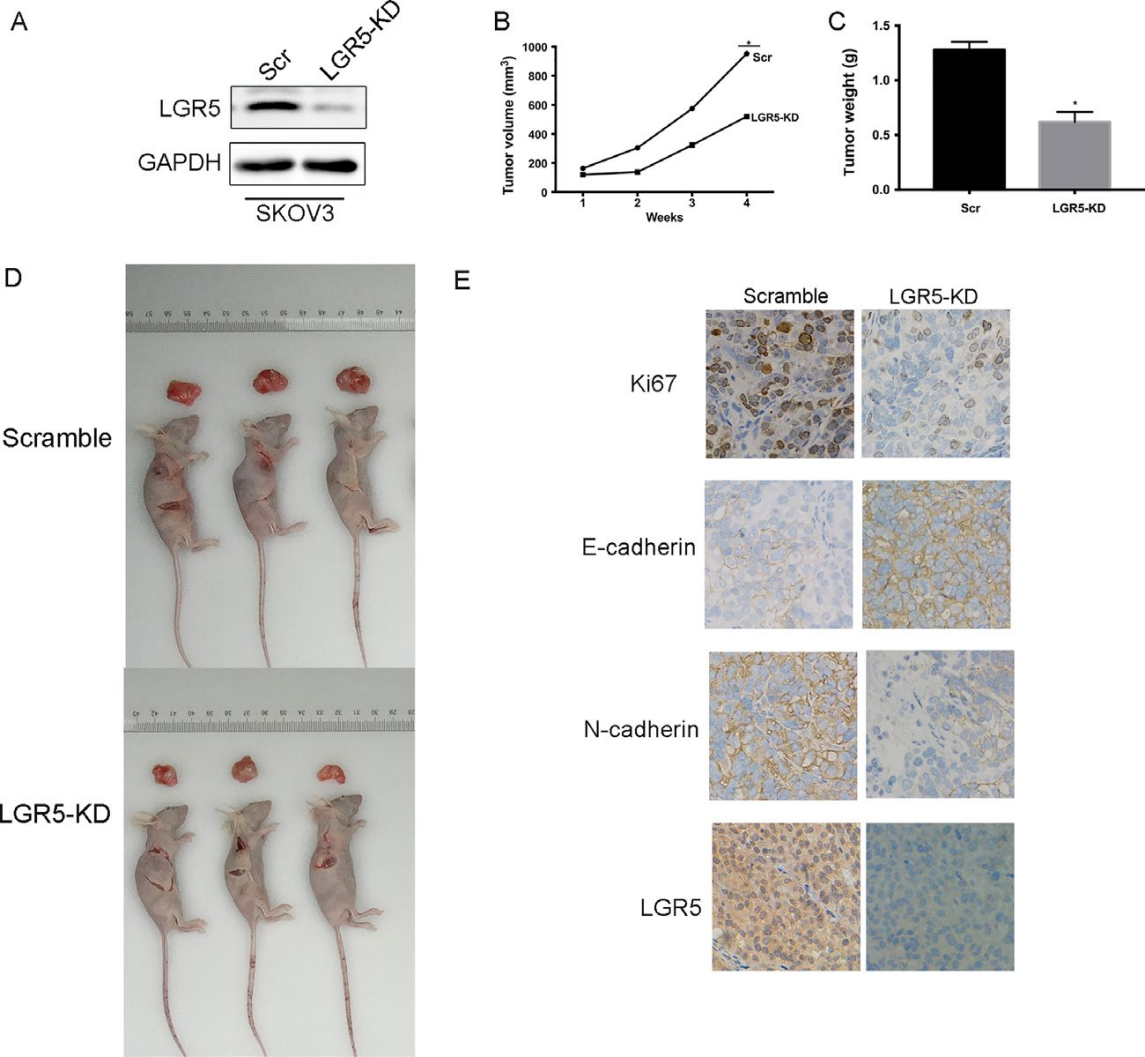
LGR5 promotes EOC progression in nude mice. (A) Western blot analysis demonstrates the effect of SKOV3 transfection. (B–C) Tumor volume and weight were decreased in the LGR5‐KD mouse model. (D) Representative data demonstrating that LGR5 knockdown (LGR5‐KD) significantly inhibited tumor growth in nude mice xenograft models. (E) Immunohistochemical staining of Ki67, E‐cadherin, and N‐cadherin, LGR5 in tumors from the LGR5‐KD and scramble control group tumors. Original magnification 200×, scale bar = 20 *μ*m.

We further tested whether knockdown of LGR5 could suppress the tumorigenicity of EOC cells in vivo, using a xenograft model in nude mice. As shown in Figure 7D, tumors formed by injection of cells treated with LGR5‐KD were significantly smaller than those treated with scrambled control. Tumor growth and tumor weight were significantly inhibited in LGR5‐KD‐injected tumors (Fig. 7B and C; *P* < 0.05), and immunohistochemical analysis showed that the LGR5‐KD group exhibited weaker expression of Ki‐67, which is a marker of proliferation (Fig. 7E), indicating that LGR5 exert oncogenic effects during epithelial ovarian cancer progression. Furthermore, in tumor xenograft experiments, the LGR5 knockdown group exhibited higher immunohistochemical staining for the epithelial marker, E‐cadherin, in addition to weaker expression of the mesenchymal‐associated molecule, N‐cadherin (Fig. 7E). These results indicate that LGR5 can promote EOC tumorigenesis and EMT in vivo.

## Discussion

LGR5, which belongs to the G protein‐coupled receptor family of proteins, is recognized as a stem cell marker of the intestinal epithelium and the hair follicle 6, 7, and has been identified as an oncogene in various cancers, including colorectal cancer 24, cervical cancer 25, neuroblastoma 26, and ovarian cancer 9. In our study, we focused on ascertaining the molecular function of LGR5 in EOC. We observed that LGR5 was overexpressed in EOC cell lines and tissues. Furthermore, upregulation of LGR5 expression was significantly correlated with patient age (*P* = 0.006), histologic type (*P* < 0.001), and metastasis (*P* = 0.025) in this malignancy. These findings strongly support a role for LGR5 in promotion of the development and progression of EOC, similar to its function in other types of carcinoma.

Our experiments also show that downregulation of LGR5 can suppress EOC cell proliferation and inhibit the tumorigenicity of ovarian cells in vitro (Fig. 3). Moreover, tumor xenograft experiments in nude mice demonstrated that LGR5 has strong tumorigenicity (Fig. 7). In contrast, the overexpression of LGR5 led to the significant promotion of ovarian cell proliferation in comparison with controls.

Previous studies have proven that LGR5 has a major role in enhancing tumor invasion and metastasis. Cell scratch‐wound and tumor cell invasion assays in vitro indicated that overexpression of LGR5 remarkably promoted the migration and invasion capacity of EOC cells, while the opposite results were observed on LGR5 knockdown. As EMT is commonly involved in cell mobility changes, and LGR5 has been identified as involved in EMT progression in breast cancer 22 and hepatocellular carcinoma 27, we investigated the expression of EMT markers and EMT‐related transcription factors by Western blotting. Our data demonstrated that the epithelial marker, E‐cadherin, was upregulated, whereas levels of the mesenchymal markers, N‐cadherin and Vimentin, were decreased when we knock‐down LGR5. In addition, level of the EMT‐related transcription factor, Snail, was downregulated in response to LGR5 knockdown. Notch signaling influences cell proliferation and apoptosis and is activated by binding to the specific ligands (Jagged1, Jagged2, Dll1, Dll3, and Dll4) 28, 29. Accumulating evidence from recent studies suggests that Notch1 signaling is involved in the process of EMT in various type of carcinoma 30, 31. In the present study, we provide evidence that LGR5 can promote ovarian cancer progression via Notch1 signaling in EOC. Taken together, our findings indicate that LGR5 expression may have a critical role in epithelial ovarian cancer tumorigenesis.

In summary, the present study demonstrates that LGR5 is overexpressed in EOC tumor samples and that dysregulated LGR5 expression, which occurs frequently in EOC, promotes tumor proliferation, metastasis, and EMT of EOC cells through the Notch1 signaling pathway. For the first time, our study examined the effects of LGR5 on the biological behavior of epithelial ovarian cancer and explored the relationship between LGR5 and Notch1 pathway in epithelial ovarian cancer. These findings suggest that LGR5 is a promising new molecular target for novel preventive and therapeutic strategy for EOC and that LGR5 expression is critical for the progression and invasiveness of EOC. Further investigations are required to fully elucidate the molecular mechanisms governing LGR5 dysregulation and its role in EOC progression. In addition, LGR5 is recognized as a stem cell marker of the intestinal epithelium and the hair follicle and Notch1 signaling also plays a fundamental role in stem cells in cell proliferation and differentiation 10. We plan to further dissect the roles of Notch1 and LGR5 in EOC, especially by exploring the role of LGR5 high cells as EOC CSCs.

## Conflict of Interest

The authors declare that they have no competing interests.
